# Efficacy of an improved absorbent pad on incontinence-associated dermatitis in older women: cluster randomized controlled trial

**DOI:** 10.1186/1471-2318-12-22

**Published:** 2012-05-29

**Authors:** Junko Sugama, Hiromi Sanada, Yoshie Shigeta, Gojiro Nakagami, Chizuko Konya

**Affiliations:** 1 Department of Clinical Nursing, Institute of Medical, Pharmaceutical and Health Sciences, Kanazawa University, Ishikawa, Japan; 2 Wellness Promotion Science Center, Institute of Medical, Pharmaceutical and Health Sciences, Kanazawa University, Ishikawa, Japan; 3 Department of Gerontrogical Nursing/Wound Care Management, Division of Health Sciences and Nursing, Graduate School of Medicine, The University of Tokyo, Tokyo, Japan; 4 School of Nursing, Kanazawa Medical University, Ishikawa, Japan

## Abstract

**Background:**

Most older adults with urinary incontinence use absorbent pads. Because of exposure to moisture and chemical irritating substances in urine, the perineal skin region is always at risk for development of incontinence-associated dermatitis (IAD). The aim of this study was to examine the efficacy of an improved absorbent pad against IAD.

**Methods:**

A cluster randomized controlled design was used to compare the efficacy of two absorbent pads. Female inpatients aged ≥65 years who had IAD and used an absorbent pad or diaper all day were enrolled. Healing rate of IAD and variables of skin barrier function such as skin pH and skin moisture were compared between the usual absorbent pad group (n = 30) and the test absorbent pad group (n = 30).

**Results:**

Thirteen patients (43.3%) from the test absorbent pad group and 4 patients (13.3%) from the usual absorbent pad group recovered completely from IAD. Moreover, the test absorbent pad group healed significantly faster than the usual absorbent pad group (p = 0.009). On the other hand, there were no significant differences between the two groups in skin barrier function.

**Conclusion:**

The test absorbent pad for older adults with urinary incontinence might be more efficacious against IAD than usual absorbent pad.

**Trial registration:**

UMIN-CTR: UMIN000006188

## Background

Incontinence-associated dermatitis (IAD) has been attracting attention in recent years [1]. IAD was described as a variety of terms including perineal skin injury [2], diaper dermatitis [3], and irritant contact dermatitis of the vulva [4] in previous studies. IAD is an inflammation of the skin that occurs when urine or stool comes into contact with perineal or perigenital skin and is associated with itchiness, pain, and dermatological symptoms that are uncomfortable and painful to affected individuals [1]. Reported prevalence rates of IAD vary at 5.6–50% [1]. In Japanese older patients with incontinence using diaper and absorbent pads, the prevalence of IAD was 17% [5]. Thorough skin cleansing and application of water-repellent skin protecting creams have been used to prevent dermal inflammation [6]. Despite these preventive care practices, however, skin impairment still commonly occurs in the elderly because decreased skin barrier function due to aging reduces the resistance of the skin to moisture exposure [7-9]. When urinary incontinence occurs during bed rest, women are more susceptible to IAD due to their anatomical structure, especially the area exposed to urine from the perineum through the coccyx to the sacrum. Focusing on IAD in older women, researchers observed an increase in moisture content and pH level of the stratum corneum of the buttock skin as a result of urinary incontinence even without diarrhea [10]. They also demonstrated a significant association between pH level of the surface of incontinence pads used and pH level of the skin and its moisture content [10].

Based on these results we expected that unless the absorbent pad that is in direct contact with the skin is modified and improved, IAD could not be managed. Moreover, Farage and colleagues demonstrated that an improved apertured film plus feminine pad for appeared to contribute less to the further development of inflammation [11]. Consequently, an incontinence absorbent pad, the dry-feel Attent S Care Pad with frontal absorbent material (Daio Paper Corporation, Tokyo, Japan) was co-developed to control IAD caused by urinary incontinence. The structure of this test absorbent pad is designed to absorb urine in the frontal area of the pad to minimize exposure of the buttocks to urine, while preventing the absorbed urine from flowing back to the pad surface. Results from older adult women with urinary incontinence demonstrated the test pad’s improved frontal absorption and backflow prevention features as compared with conventional products [12]. The use of the test absorbent pad on individuals with IAD is expected to result in early cure without worsening the skin condition. However, its efficacy has yet to be demonstrated.

The aim of this study was to examine the efficacy of the test absorbent pad against IAD. We hypothesized that the skin condition of older adult women with IAD would improve faster with the use of the test absorbent pad versus usual pad as control.

## Methods

### Study design

A cluster randomized controlled design was used to compare the efficacy of the two absorbent pads. The study complied with CONSORT [13]. The randomization occurred at unit level to avoid contamination and the units were randomized using opaque envelopes by a researcher not involved in the study. An independent researcher randomized ten units at a 500-bed geriatric medical hospital in Ishikawa, Japan to either control or experimental group, 5 units for usual care and 5 for intervention care.

Effective blinding was not possible for the intervention administrator because the appearance of the test absorbent pad clearly differed from that of control pad. Therefore, the skin evaluator who was not involved in the intervention served as blinded outcome evaluator.

### Participants

Inclusion criteria were adult women aged ≥65 years, diagnosed with IAD, and full-time user of diapers (including absorbent pads) because of urinary incontinence. No criteria were specified regarding the type of incontinence. Exclusion criteria were diagnosis of a skin disorder other than IAD in the buttocks area such as pressure ulcers that required special medical care other than the selection of absorbent pad, diarrhea, poor general medical status, and scheduled to be discharged within ≤1 week.

The head ward nurse was asked to compile a list of women using absorbent pads and diapers for urinary incontinence. On the first day of the study, 10 research assistants who had undertaken skin observation training were split into pairs. They observed the buttock skin condition of the listed patients at diaper change. If IAD was identified on the buttocks by the trained research nurses, the individual was eligible for recruitment into the study. The allocation period was from August to September 2007. Patients were followed until they fully recovered from IAD for a maximum of 1 week after recruitment.

### Intervention

**Figure 1:**
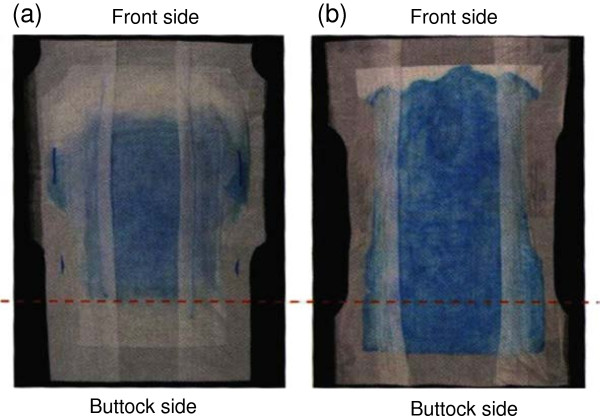
**Diffusion of urine on test absorbent pad (a) and control absorbent pad (b).** Diffusion of urine of the test pad is narrower than that of control pad and the test absorbent pad prevented leakage into the buttocks area.

Patients in the intervention group wore the test absorbent pad and a diaper throughout the day from 09:00 to 20:00. The width of the absorbent material in the frontal area of the test pad is 23 cm, designed to absorb the urine on the spot at the urinary excretion point (Figure 1). Since the absorbent material, a combination of an absorbent polymer and pulp, is located only in the frontal area of the pad, the urine is less likely to leak into the buttocks area. In addition, the slit in the urinary excretion point and the flexed convex surface of the pad fit in the perineal region, which also prevents leakage into the buttocks area. Furthermore, a second sheet is embedded between the top sheet and the absorbent material to prevent absorbed urine from flowing back to the surface. During the night (from 20:00 to 09:00), patients in the intervention group wore the hospital-standard pad (Urine Absorbing Pads-Big; Iwatsuki Co. Ltd., Tokyo, Japan) and a diaper. The reason for this switch pad was that the allowance volume of the test pad. The allowance volume of the test pad was not adequate to the care regimen for changing the incontinence pad during the night in the test hospital. Control group patients, on the other hand, wore the standard pad and diaper at all times.

The staff of each unit changed the diapers. The frequency, procedure, and skin care routines of changing diapers in both groups followed the hospital’s care standards.

### Main outcomes

Skin observation and photographs of the buttock area were undertaken for both the test pad group and the control group every day at 20:00 by 3 researchers. The main outcome observation period was 1 week from recruitment and ended when IAD was cured.

The main outcome indicators were cure and severity of IAD. Severity was assessed by IAD Skin Condition Assessment Tool [1] consisting of three categories such as areas of skin breakdown, skin redness, and skin erosion. Areas of skin breakdown and skin redness are rated from 0 to 3, and skin erosion from 0 to 4. Higher scores indicate higher levels of severity. Observational assessments were made based on the skin that was in direct contact with the urinary absorbent pad. In this study, IAD was rated based on the buttock pictures, performed by a researcher who was not aware of the test pad and control group allocation. A total IAD score of zero was considered as cured.

### Secondary outcomes

Moisture content of the stratum corneum of the coccyx skin and skin pH were measured at the time of recruitment and at the end of the study. Moisture content of the stratum corneum was measured by Corneometer® CM825 (Courage + Khazaka GmbH, Cologne, Germany). The measurement depth for moisture content was 30–40 μm of the stratum corneum. The measuring principle of the corneometer is based on electrostatic capacitance method, which measures the change in the dielectric constant associated with change in moisture content of the skin. Measurement accuracy was ±3%, and the measurement values were expressed as proportional values from 0 to 120, with 20 and 120 being indicative of skin dryness and skin hydration, respectively. This method allows immediate and noninvasive measurement of skin moisture content. This corneometer has been used to measure the moisture content of the stratum corneum in various dermatological conditions and its reliability and adequacy have been demonstrated [14]. Skin pH value was assessed by Skin-pH-meter® PH900 (Courage + Khazaka GmbH, Cologne, Germany). As for the measuring principle, measuring and reference electrodes are placed into a single combined electrode that is encased in a glass membrane and attached to the skin surface through ion-permeable membrane adjacent to the glass membrane. This allows direct measurement of pH value from the skin surface. The reliability and adequacy of the pH meter have been demonstrated [15]. The moisture content of the stratum corneum and the pH value were measured three times each; the mean values were used in the analysis.

### Statistical analysis

Time-to-healing curves were drawn for the test pad group and control group using the Kaplan-Meier method. Time-to-healing curves of the two groups were compared by log-rank test. Cox proportional hazard analysis was used to assess predictors of healing outcomes. The relation between type of absorbent pad and change in symptoms (improved versus unchanged/worsened) was examined by *χ*^2^ test. Moisture content of the stratum corneum and skin pH value at recruitment and at the end of the study were compared by *t*-test. SPSS Version 17 was used for statistical analysis; significance level was set to 0.05.

### Ethical considerations

The study objective and procedures were explained to the study subjects or to their family members, and written informed consent was obtained. This study was approved by the Ethics Committee of the Graduate School of Medicine, University of Tokyo (#1817).

## Results

### Participant flow

**Figure 2:**
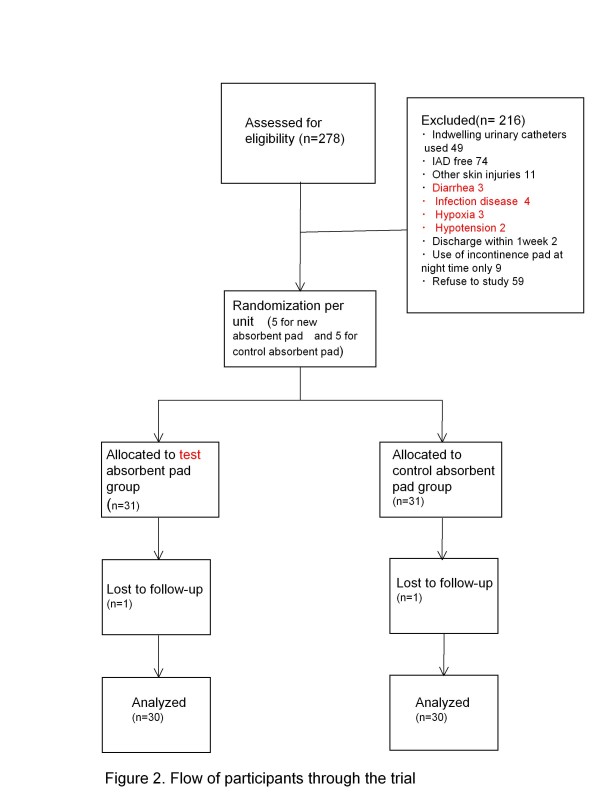
Patients flow diagram.

The flow of participants is depicted in Figure 2. At the study site there were 278 female inpatients with urinary incontinence who were aged ≥65 years. The following patients were excluded: no symptoms of IAD (n = 74); those using indwelling urinary catheters (n = 49); skin problems other than IAD in the buttock area (n = 11); diarrhea (n = 3); infection disease (n = 4); hypoxia (n = 3); hypotension (n = 2); scheduled to be discharged within ≤1 week (n = 2); use of incontinence pad at night time only (n = 9); and no consent to participate in the study from the patient or family member (n = 59). Sixty-two patients who participated in the study were assigned to two groups: the study group (n = 31) and the control group (n = 31). One patient from each group subsequently withdrew from the study; therefore the final number of study subjects was 30 in the study group and 30 in the control group.

### Baseline data

The patients’ characteristics are shown in Table 1. There was no significant difference between the two groups in age, BMI, disease, and degree of independence in daily living. IAD skin conditions for the two groups are shown in Table 2. No significant difference was observed in the area of skin breakdown, intensity of skin redness, and skin erosion. Skin erosion was not observed in any of the patients at the time of recruitment. There was no significant difference in the moisture content of the stratum corneum between the test absorbent pad group and the control group (P = 0.959). Likewise, no significant difference was found in skin pH between the test absorbent pad group and control group (P = 0.423) (Table 2).

**Table 1:** Patient characteristics

**Characteristics**	**Control group**	**Test pad group**	**P value**
	**(n = 30)**	**(n = 30)**	
Age in years, mean(SD)	84.5(8.4)	84.9(7.3)	0.832
BMI,mean(SD)	17.7(3.2)	17.3(3.1)	0.650
Disease,n(%)			0.213
CVD^1^	19(63.3)	23(76.7)	
Dementia	3(10.0)	5(6.7)	
Heart disease	2(6.7)	0(0.0)	
Others	6(20.0)	2(6.6)	
Rank of dependecy on caregivers for daily living^2^, n(%)			0.267
A	0(0.0)	0(0.0)	
B	7(23.3)	12(40.0)	
C	23(76.7)	18(60.0)	

SD, standard deviation; BMI, body mass index; CVD, cerebrovascular disease.

1 CVD: Cerebrovascular disease.

2 group A elderly were house-bound, Group B elderly spent majority of the time in wheelchair, and Group C elderly were bedridden.

**Table 2:** IAD skin condition, moisture content, and skin pH at baseline

**Skin condition**	**Control group**	**Test pad group**	**P value**
	**(n = 30)**	**(n = 30)**	
Areas of skin break down, n(%)			
Small area	3(10.0)	6(20.0)	0.27
Moderate area	9(30.0)	12(40.0)	
large area	18(60.0)	12(40.0)	
Skin redness, n(%)			
No	0(0.0)	1(3.3)	0.22
Mild redness	15(50.0)	20(66.7)	
Moderate redness	13(43.3)	9(30.0)	
Severe redness	2(6.7)	0(0.0)	
Erosion			
None	30(100.0)	30(100.0)	
Skin moisture, mean (SD)	67.6(28.3)	67.2(27.0)	0.96
Skin pH, mean (SD)	6.9(0.7)	6.8(0.6)	0.42

Skin condition was evaluated by IAD Skin Condition Assessment Tool (Gray 2007).

### Outcome measures

**Figure 3:**
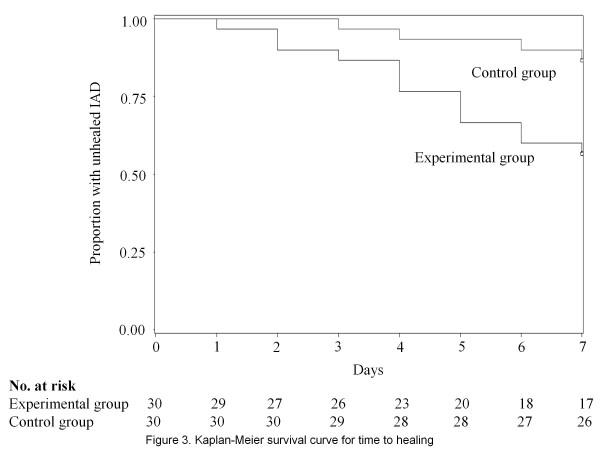
Healing curve.

Thirteen patients (43.3%) from the test absorbent pad group and 4 patients (13.3%) from the control group recovered completely from IAD, a statistically significant difference (P = 0.010). On the other hand, 13 patients (43.3%) in the test absorbent pad group and 15 patients (50.0%) in the control group showed no change or an increase in the scores (Table 3). The test absorbent pad group recovered significantly faster from IAD, as indicated by the Kaplan-Meier curve (*P* = 0.009; log-rank test) (Figure 3). Cox regression analysis revealed that use of the test absorbent pad was a significant independent predictor of the primary outcome (adjusted hazard ratio [HR], 0.283; 95%CI, 0.089–0.896; P = 0.032) regardless of age and BMI (Table 4).

**Table 3:** IAD total score change

**Change**	**Control group**	**Test pad group**
	**(n=30)**	**(n=30)**
Process, n(%)		
Healing	4(13.3)	13(43.3)
Improved	11(36.7)	4(13.4)
No chage	9(30.0)	7(23.3)
Deteriorated	6(20.0)	6(20.0)

Increasing and decreasing total score during the observational period indicates IAD deterioration and improvement; zero means complete healing.

**Table 4:** Cox regression analysis: predictors of healing

**Variables**	**Adjusted hazard ratio**	**95% CI**	**P value**
Use of test absorbent pad	0.283	0.089-0.896	0.032
Age	0.959	0.903-1.018	0.170
BMI	0.980	0.581-1.130	0.785
Initial severity of IAD	0.67	0.410-1.100	0.12

There was no significant difference in the moisture content of the stratum corneum between the test absorbent pad group and the control group at the end of study period (P = 0.823). Likewise, no significant difference was found in skin pH between the test absorbent pad group and the control group (P = 0.761) (Table 5).

**Table 5:** Moisture content and skin pH at end of the study period

	**Control group**	**Test pad group**	**P value**
Skin condition	(n=30)	(n=30)	
Skin moisture, mean (SD)	66.4(29.5)	64.8(25.9)	0.82
Skin pH, mean(SD)	6.6(0.5)	6.5(0.5)	0.76

## Discussion

The present cluster randomized controlled trial examined the hypothesis that the skin condition of older women improves faster with the use of the test absorbent pad than with conventional products. We observed that significantly faster recovery could be achieved using the test absorbent pad.

IAD is the result of damage to the skin when exposed to stool or urine. Prolonged exposure to higher than normal levels of water results in maceration, barrier breakdown, disruption of intercellular lamellar lipid bilayers, degeneration of corneodesmosomes, and formation of amorphous regions within the intercellular lipid [3]. Moreover, when urine is excreted into the absorbent pad, urea in the urine is broken down to ammonia, which tends to tip the scale towards the alkaline side, thereby irritating the skin. Chemical and physical stimuli due to frequent cleansing enhance skin permeability and decrease skin barrier function. These factors weaken the skin, which then triggers IAD [6]. Randomized, controlled trials (RCT) investigating effective management of IAD through skin care programs or the use of incontinence pads have been previously conducted. As for skin care programs, there are a number of studies particularly on skin cleansers, skin protectors, and moisturizers [7-9]. However, even after these treatments, patients develop IAD. For this reason, we came to recognize the importance of improving the incontinence absorbent pad that is in direct contact with the skin. In terms of incontinence absorption pads, studies have reported improved skin condition with the use of pads containing absorbent polymers [16,17]. Even with the use of absorbent polymers, however, there remained a significant association between the pH level of the incontinence pad surface and that of the skin, which is a risk factor for developing IAD [16]. To prevent alkalinization of the skin, Beguin *et al.*[18] developed an absorbent pad that consisted of an absorbent layer with a specially formulated pH-controlling fiber and a highly breathable material in the side panel that can maintain a weakly acidic skin pH. In the clinical evaluation of that pad, a case series of 12 elderly patients with IAD was studied, and full recovery was reported in 8 patients on day 21 of use. However, since the study did not have a control group, and its results were inclusive of natural healing process, the true efficacy of the pad in curing IAD is unknown.

In this study we were able to demonstrate that significantly faster recovery could be achieved using the test absorbent pad. Our previous studies demonstrated that effective frontal absorption of the test absorbent pad and its mechanism to prevent urine from seeping back to the surface significantly reduced the pH level of the pad [12]. This condition is believed to have facilitated full recovery from IAD. However, although a significant difference was observed in the healing rate, the proportion of patients with no change or aggravation in skin condition was similar for both the test absorbent pad group (43.3%) and the control group (50.0%). In addition, there were no significant differences in the moisture content of the stratum corneum of the skin on the coccyx and the skin pH level. The reason for these may be due to the study design that the test absorbent pad group used the control group’s absorbent pad at night, which may have had an influencing effect. The reason for this switch pad was that the allowance volume of the test pad.

A limitation is that this study targeted bedridden older women and therefore its findings do not apply to elderly people leading sedentary or ambulatory lifestyles. Our sample size was not based on the power analysis.

## Conclusions

Using the test incontinence absorbent pad that incorporates frontal absorption and a backflow prevention mechanism, a significantly faster, full recovery was observed among older women with urinary IAD compared with those in the control group using a conventional pad. Our results suggest good efficacy of the test absorbent pad in facilitating recovery from IAD.

## Competing interests

Test pads were generously donated by Daio Paper Corporation, Tokyo, Japan. The study and writing of the article was done without any financial support by any company. The company was not informed before, during, or after the study and writing of the article.

## Authors’ contributions

JS & HS (scientific oversight) and YS (principle investigator) designed the study. The research protocol was refined by JS and CK. GN was responsibile for statistical analysis. JS wrote the first draft of the manuscript and was responsible for revisions. All authors read and approved the final manuscript.

## Pre-publication history

The pre-publication history for this paper can be accessed here:

http://www.biomedcentral.com/1471-2318/12/22/prepub
